# Oral Exposure to Bisphenol A Increases Dimethylbenzanthracene-Induced Mammary Cancer in Rats

**DOI:** 10.1289/ehp.11751

**Published:** 2009-01-07

**Authors:** Sarah Jenkins, Nandini Raghuraman, Isam Eltoum, Mark Carpenter, Jose Russo, Coral A. Lamartiniere

**Affiliations:** 1 Department of Pharmacology and Toxicology; 2 UAB Comprehensive Cancer Center and; 3 Department of Pathology, University of Alabama at Birmingham, Birmingham, Alabama, USA; 4 Department of Mathematics and Statistics, Auburn University, Auburn, Alabama, USA; 5 Breast Cancer Research Laboratory, Fox Chase Cancer Center, Philadelphia, Pennsylvania, USA

**Keywords:** apoptosis, bisphenol A, mammary cancer, proliferation, steroid receptor coactivators

## Abstract

**Background:**

Bisphenol A (BPA) is widely used in the manufacture of polycarbonate plastics, including infant formula bottles.

**Objectives:**

Based on the reported endocrine disruptor activity of this polyphenol, we hypothesized that exposure to BPA early in life would elicit developmental changes in the mammary tissue and cause a predisposition for mammary cancer.

**Methods:**

We exposed neonatal/prepubertal rats to BPA via lactation from nursing dams treated orally with 0, 25, and 250 μg BPA/kg body weight/day. For tumorigenesis studies, female offspring were exposed to 30 mg dimethylbenzanthracene (DMBA)/kg body weight at 50 days of age.

**Results:**

The combination of DMBA treatment with lactational exposure to BPA demonstrated a dose-dependent increase in mammary tumor multiplicity and reduced tumor latency compared with controls. In the absence of DMBA treatment, lactational BPA exposure resulted in increased cell proliferation and decreased apoptosis at 50 but not 21 days postpartum (shortly after last BPA treatment). Using Western blot analysis, we determined that steroid receptor coactivators (SRCs) 1–3, Akt, phosphorylated Akt, progesterone receptor A (PR-A), and erbB3 proteins were significantly up-regulated at 50 days of age.

**Conclusions:**

The data presented here provide the first evidence that maternal exposure to BPA during lactation increases mammary carcinogenesis in a DMBA-induced model of rodent mammary cancer. Changes in PR-A, SRC 1–3, erbB3, and Akt activity are consistent with increased cell proliferation and decreased apoptosis playing a role in mammary cancer susceptibility. These alterations provide an explanation of enhanced mammary carcinogenesis after lactational BPA exposure.

Breast cancer etiology attributed to hereditary causes is low, accounting for an estimated 5–15% of all diagnosed cases (Colditz and Frazier 1995). Extrinsic factors, such as environment and lifestyle, may play substantial roles in the high incidence of breast cancer. Routine exposures to certain environmental contaminants, especially those with the ability to alter endocrine signaling, are currently being scrutinized for their potential roles in breast cancer causation. One such example can be found in the synthetic estrogen diethylstilbestrol (DES). Women were prescribed DES from 1947 until 1971 to prevent miscarriages. Not only was the drug therapeutically ineffective, but reports have established that women exposed *in utero* to DES showed an increased incidence of a rare type of vaginal cancer and an increased risk of developing breast cancer (Herbst et al. 1999; Troisi et al. 2007). These effects, occurring more than a decade after the original exposure, have caused greater public awareness of the role of early endocrine-disruptor exposure in lifetime breast cancer susceptibility. Likewise, recent media attention has focused on another common environmental endocrine disruptor, bisphenol A (BPA), as a potentially harmful chemical because of its widespread exposure to humans and reported endocrine-disruptor activity.

BPA is ubiquitous in modern society. The main source of BPA stems from its widespread use to manufacture polycarbonate plastic and epoxy resins used in beverage and food containers, infant formula bottles, canned food liners, and some dental sealants, among other uses. However, the bonds that connect the BPA monomers are weak and highly subject to degradation even with normal use (Le et al. 2008). Factors such as time, higher temperatures, and pH extremes accelerate this process (Kang et al. 2003).

Calafat et al. (2005) found that 95% of adults surveyed (*n* = 394) had detectable concentrations of total (free plus conjugated) urinary BPA. In a pilot study of the concentrations of a panel of environmental chemicals in the urine of young girls, Wolff et al. (2007) reported similar findings. They reported total BPA concentrations ranging from below the limit of detection (0.3 μg BPA/L) to 54.3 μg BPA/L, with an average value of 2.0 μg BPA/L (3.0 μg BPA/g creatinine). A large-scale investigation supported these previous studies (Calafat et al. 2008), finding detectable concentrations of total BPA in 93% of > 2,000 participants, averaging 2.6 μg BPA/L. Furthermore, concentrations of total BPA increased with decreasing age. Children (6–11 years of age) had the highest concentrations of total BPA [least square geometric mean (LSGM), 4.5 μg BPA/L], significantly higher than those of adults (LSGM, 2.5 μg BPA/L) (Calafat et al. 2008). BPA has been found to leach from polycarbonate infant formula bottles and as free and conjugated forms in breast milk, suggesting several sources for early BPA exposure and creating the concern that long-lasting adverse health effects may arise as a consequence (Brede et al. 2003; Sun et al. 2004; Ye et al. 2006).

Several studies using animal models have linked early exposure to BPA with developmental and reproductive abnormalities in both sexes (Ho et al. 2006; Howdeshell et al. 1999). Perinatal exposure to BPA at 0.1 or 1.2 mg/kg body weight (bw) per day through drinking water significantly increased body weight, altered estrous cyclicity, and significantly lowered plasma concentrations of luteinizing hormone (Rubin et al. 2001). Perinatal exposure to BPA at 250 ng/kg bw/day through an osmotic pump caused significant alterations in the mammary gland, including an increased number of terminal end buds (TEBs), decreased apoptosis in TEBs, an increased percentage of cells expressing the progesterone receptor (PR), and increased lateral branching (Munoz-de-Toro et al. 2005). With gestational exposure alone, BPA advanced puberty and increased the number of terminal ducts, TEBs, alveolar buds, and preneoplastic lesions in the mammary gland (Markey et al. 2001; Murray et al. 2007). These and other studies provide a substantial amount of evidence to warrant further investigation into the potential deleterious effects caused by early exposure to BPA, especially in terms of mammary carcinogenesis.

We sought to determine whether early exposure to BPA could accelerate mammary carcinogenesis in a dimethylbenzanthracene (DMBA) model of rodent mammary cancer. We treated lactating dams with low concentrations of BPA: 25 μg/kg/day, which is one-half of the U.S. Environmental Protection Agency’s (EPA) daily oral reference dose of 50 μg/kg/day (BPA 25 group), and 250 μg/kg/day, which is 200-fold less than the lowest observed adverse effect level (LOAEL) of 50 mg/kg/day (BPA 250 group) (National Toxicology Program 1982). Thus, the present study provides the first evidence that oral administration of low concentrations of BPA to lactating dams can increase mammary cancer susceptibility of the female offspring in a DMBA-induced model. Our data suggest that the mechanism of action behind this response is mediated through increased cell proliferation and decreased apoptosis and centers on an up-regulation of steroid receptor coactivator (SRC) proteins, erbB3, and Akt signaling in the mammary gland.

## Materials and Methods

### Animals

Animal care and use were conducted according to established guidelines approved by the Institutional Animal Care and Use Committee at the University of Alabama at Birmingham. Animals were treated humanely and with regard for alleviation of suffering. All animals were housed in a temperature- controlled facility with a 12 hr light/dark cycle. Female Sprague Dawley CD rats (Charles River, Raleigh, NC) were bred and observed for the presence of sperm. Sperm-positive females were separated, housed in polypropylene cages with glass water bottles (both polycarbonate/BPA free), and fed phytoestrogen-free AIN-93G pelleted diet (Harlan Teklad Global Diets, Wilmington, DE). On day 2 post-partum, all female offspring (5–8) and enough males to yield 10 offspring/litter were retained. Beginning on postpartum day 2 and continuing through postpartum day 20, the lactating dam of each litter was intragastrically gavaged with sesame oil vehicle (SO) or with BPA at 25 μg/kg/day (BPA 25) or 250 μg/kg/day (BPA 250) on Monday through Friday only (a total of 15 treatments). Because dam treatment for lactational exposure results in a single exposure group per treatment, we used only one offspring from each litter in each experiment.

### Chemicals

We purchased BPA and sesame oil from Sigma Chemical Co. (St. Louis, MO). We purchased antibodies to erbB2, Bax, phosphorylated Bad, Bcl-2, caspase 2, caspase 3, and cleaved caspase 3 from Cell Signaling (Danvers, MA); antibodies to erbB3, estrogen receptor-α (ER-α), and PR-A and PR-B from Santa Cruz Biotechnology (Santa Cruz, CA); and antibodies to SRC-1, SRC-2/TIF2, and SRC-3/AIB1 from Becton, Dickenson and Company (Franklin Lakes, NJ).

### Tumorigenesis study

At 50 days of age, one female offspring from each litter of each treatment group was given a single gavage of DMBA 30 mg/kg bw. This dose results in a low number of mammary tumors and allows chemicals that predispose for mammary cancer to increase the number of mammary adenocarcinomas (Brown et al. 1998). Because dam treatment for lactational exposure results in a single exposure group per treatment, we used only one offspring from each litter in each experiment. There were 32, 34, and 24 female offspring in the SO, BPA 25, and BPA 250 groups, respectively, all derived from individual litters. We palpated animals twice weekly to monitor tumor development and recorded data on palpable tumor latency, location, tumor burden, and multiplicity. Animals underwent necropsy at 12 months of age or when tumor burden exceeded 10% of body weight. All tumors and gross lesions were dissected out, fixed in formalin, and embedded in paraffin, and sections were prepared for pathologic evaluation. Coded slides were classified as to tumor type, tissue of origin, and degree of invasiveness by a board-certified pathologist (I.E.). Histopathologic characterization of mammary neoplastic lesions included carcinoma grade, proliferation index, and malignancy evaluations, as described by Meyer et al. (2005).

### Mammary dissections

When animals were 21 and 50 days of age, we processed two sets of identically treated rats using ketamine/xylazine anesthesia for live collection of mammary glands. The fourth abdominal mammary glands were collected from each rat; one set of the mammary glands was snap frozen in liquid nitrogen for immunoblotting (*n* = 8 per treatment group), and the contralateral glands were fixed in formalin and embedded in paraffin (*n* = 5 per treatment group). At 50 days of age, all females were killed during the estrous phase.

### Hormone concentration

At 50 days of age, female offspring lactationally exposed via dams treated with BPA 250 or an equal volume of sesame oil were decapitated and trunk blood was collected. We measured serum 17β-estradiol and progesterone concentrations using radioimmunoassays (Diagnostic Systems Laboratories, Webster, TX) as described by the manufacturer. All samples were run in duplicate, with eight samples per group, by J. Mahan (Obstetrics and Gynecology Department, University of Alabama at Birmingham).

### Immunohistochemical staining

To determine cell proliferation, tissue blocks were sectioned at 5 μm and placed on glass slides. The slides were deparaffinized and rehydrated through xylene and graded alcohol washes. Slides were boiled in citrate buffer for 15 min, incubated in hydrogen peroxide to quench endogenous peroxidases, and blocked using the appropriate serum. We incubated the slides in Ki-67 primary antibody (Dako, Glostrup, Denmark) overnight in a humidified chamber. After incubating the tissue sections in the appropriate conjugated secondary antibody, we employed the use of the ImmPRESS kit (Vector Laboratories, Burlingame, CA). Positively stained cells were visualized by incubating the tissue sections with 3,3′-diamonobenzidine and counterstained with hematoxylin. Tissue sections were dehydrated with graded alcohols, cleared with xylene, and mounted with a glass coverslip. We used five biologically distinct samples derived from individual litters, and counted a minimum of three TEB structures per slide. We assessed cell proliferation by noting intense nuclear staining for Ki-67 protein.

### Apoptosis assay

We determined the rate of apoptosis using the ApopTag Plus Peroxidase *In Situ* Apoptosis Detection kit (Chemicon International, Billerica, MA) according to the manufacturer’s protocol. This method detects the apoptotic cells by the indirect TUNEL (terminal deoxynucleotidyl transferase dUTP nick end labeling) method. Cells that stained positively and exhibited morphologic characteristics of apoptosis were counted as positive. We used five biologically distinct samples per treatment and counted a minimum of three TEB structures per slide.

### Immunoblotting

We diced whole mammary glands and homogenized them in RIPA lysis buffer (Pierce Biotechnolgy, Rockford, IL) using a pestle and sample-grinding kit (GE Healthcare Inc., Piscataway, NJ). After homogenization, the samples were centrifuged for 20 min at 16,000 × *g* at 4°C. We quantified protein lysate recovered from the residual tissue using the Bradford assay (Bio-Rad Laboratories, Hercules, CA). Equal protein content (40 μg) was loaded onto precast sodium dodecyl sulfate (SDS) Tris-HCl 4–20% polyacrylamide gels (Bio-Rad). Proteins were wet transferred to a nitrocellulose membrane overnight. The membrane was blocked at room temperature, and the primary antibody was added and incubated overnight at 4°C. Secondary antibody and chemilume (Pierce Biotechnology, Rockford, IL) were added and protein expression was visualized using film exposures. We assessed densitometry patterns using Quantity One computer software (Bio-Rad). We used positive protein controls purchased from the supplier for the corresponding antibodies and Kaleidoscope Precision Plus protein and prestained SDS-PAGE Broad Range standards (Bio-Rad) to identify the protein of interest.

### Statistical methods

We analyzed the time-to-event data [e.g., time-to-first-tumor (latency) and time-to-sacrifice (tumor burden)] using the LIFETEST and LIFEREG procedures in SAS (SAS Institute Inc., Cary, NC). We first estimated survival functions for each group using the Kaplan-Meier method; we then compared these across the three groups using the Wilcoxon log-rank test, and parametrically using survival regression analysis (Collette 2003). Those animals that had not developed a tumor by the end of the study or that were sacrificed were censored, with either the sacrifice time or the end of study treated as censoring times. We analyzed tumor multiplicity data with the GENMOD (generalized linear models) procedure in SAS using generalized Poisson regression on the tumor appearance rates (assuming a negative binomial distribution) (McCullagh and Nelder 1989). Because there was a positive correlation between number of tumors and number of days in the study, we performed the tests on multiplicity after adjusting for the number of days each animal was in the study. Values for multiplicity are expressed as mean ± SE. For cell proliferation and apoptosis, the resulting values (stained vs. unstained cells) were used to construct a contingency table. We tested Western blots for equal variance using a two-sample *F*-test, and we used the appropriate (assuming equal or unequal variance) two-sample *t*-test. We considered *p*-values ≤ 0.05 to be statistically significant.

## Results

Female rats in the BPA 25 and BPA 250 groups had similar body weights compared with the SO group at 2, 7, 14, 21, 35, 50, and 100 days of age. Puberty, as measured by the age at vaginal opening, was not significantly altered, occurring on days 31.39 ± 0.24, 31.15 ± 0.21, and 31.88 ± 0.14 for SO, BPA 25, and BPA 250 groups, respectively. At 50 days of age, serum 17β-estradiol concentration was 34.4 ± 2.7 and 23.6 ± 4.7 pg/mL for the SO and BPA 250 groups, respectively. Circulating progesterone concentrations were 12.5 ± 2.8 and 9.9 ± 1.9 ng/mL in 50-day-old rats in the SO and BPA 250 groups, respectively. Neither of these findings was significantly different. We investigated estrous cyclicity in adult female offspring from all three treatment groups for 19 consecutive days, starting at 7–8 weeks of age, and found no significant difference.

### DMBA-induced mammary carcinogenesis

**Figure 1:**
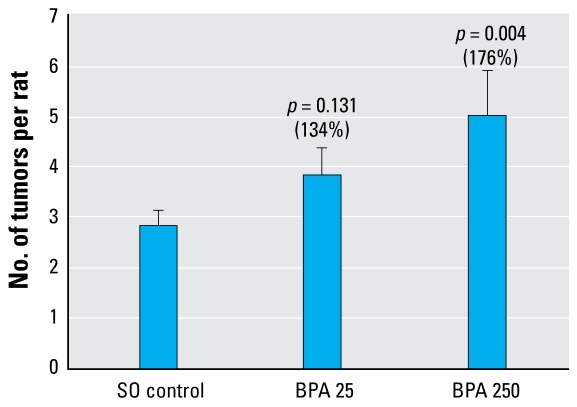
DMBA induced mammary tumors (mean ± SE) in rats exposed lactationally to BPA. *p*-Values reflect comparison with control.

Female rats in the SO, BPA 25, and BPA 250 groups gavaged at day 50 with DMBA exhibited a BPA dose-dependent increase in mammary tumors: 2.84 ± 0.31, 3.82 ± 0.43, and 5.00 ± 0.88 mammary tumors per rat, respectively (Figure 1). After adjusting for the length of time on the study (time before sacrifice), we found that the BPA 250 group had a significantly greater number of tumors per rat than did the control group (*p* = 0.004). Treatment with BPA also significantly reduced tumor latency, with median tumor latencies of 65, 53 (*p* = 0.058), and 56.5 (*p* = 0.025) days for 0, BPA 25, and BPA 250, respectively. Although the BPA 25 group had a similar decrease in latency and an increase in tumor multiplicity (*p* = 0.131), neither of these changes reached statistical significance. Histopathologic evaluation of these tumors revealed no changes in the carcinoma score, tumor burden, or length of time in the study for any treatment group (data not shown).

### Apoptosis and cell proliferation

**Figure 2:**
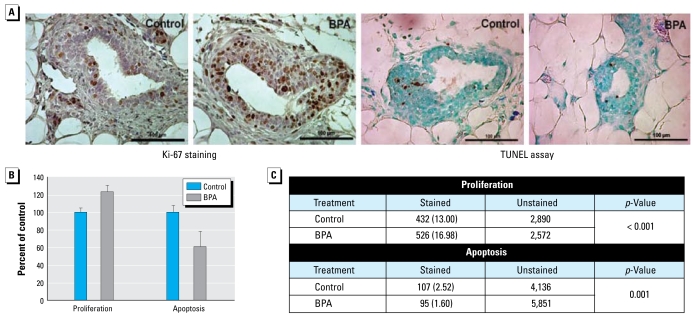
Cell proliferation and apoptosis in mammary glands of 50-day-old BPA 250 rats. (*A*) Ki-67 expression as an indicator of cell proliferation (left) and the TUNEL assay as measure of apoptosis (right). TEBs from five biologically distinct samples (*n* = 5) were analyzed per treatment. Magnification, 40×; bar = 100 μm. (*B*) Index values (mean ± SE) as a percentage of the control group. (*C*) Results [no. (%)] of cell proliferation and apoptosis analyses.

Previous reports have shown that exposure to BPA during the perinatal period through subcutaneously implanted pumps can lead to long-lasting effects on cell turnover (Durando et al. 2007; Munoz-de-Toro et al. 2005). Therefore, we assessed the rate of cellular proliferation and apoptosis in the structure most susceptible to mammary carcinogenesis, the TEB. Interestingly, cell proliferation in TEBs of 50-day-old but not 21-day-old rats was significantly increased in those animals lactationally exposed to BPA 250 (*p* < 0.001; Figure 2). Equally important, apoptosis was significantly decreased in the mammary gland of 50-day-old but not 21-day-old BPA 250 rats (*p*= 0.001).

### Akt and phosphorylated Akt (pAkt)

**Figure 3:**
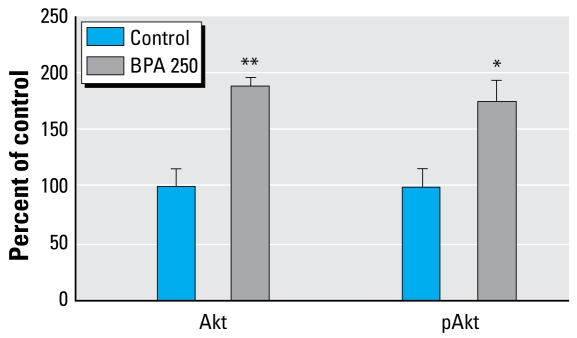
Akt and pAkt protein expression in mammary glands of 50-day-old SO controls and BPA 250 rats detected by Western blot analysis [see also Supplemental Material, Figure 1 (http://www.ehponline.org/members/2009/11751/suppl.pdf)]. For each treatment group, *n* = 8. Values shown are mean density ± SE as a percentage of control group. *p= 0.05, and **p= 0.001, compared with control.

Because of the statistically significant decrease in apoptosis in adult BPA 250 rats, we measured the expression of several proteins commonly linked to apoptosis. At 50 days of age, Akt (*p* = 0.001) and pAkt (*p* = 0.050) were significantly different between exposure groups, with each protein showing nearly a 2-fold increase over control expression levels [Figure 3; see also Supplemental Material, Figure 1 (available online at http://www.ehponline.org/members/2009/11751/suppl.pdf)]. Expressions of Bad, phosphorylated Bad, Bcl-2, caspase 2, caspase 3, and caspase 9 were not significantly different. Furthermore, none of these proteins was differentially regulated at 21 days of age, suggesting that protein expression and activity of Akt were latent effects from BPA exposure.

### Sex steroid receptors, SRCs, and growth factor receptors

**Figure 4:**
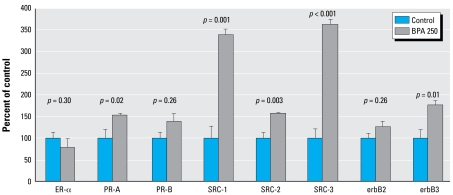
ER-α, PR-A, PR-B, SRC-1, SRC-2, SRC-3, erbB2, and erbB3 protein expression in mammary glands of 50-day-old SO control and BPA 250 rats detected by Western blot analysis [see also Supplemental Material, Figure 1 (http://www.ehponline.org/members/2009/11751/suppl.pdf)]. For each treatment group, *n* = 8. Values shown are mean density ± SE as a percentage of the control group.

Given the statistically significant increase in cell proliferation in mammary TEBs of 50-day-old offspring exposed lactationally to BPA, we assessed proteins implicated in cellular proliferation. Primarily because of BPA’s reported weakly estrogenic effects, we initially focused on sex steroid receptors. We found that BPA-exposed females exhibited an up-regulation of PR-A of 54% compared with control [*p* = 0.02; Figure 4; see Supplemental Material, Figure 1 (http://www.ehponline.org/members/2009/11751/suppl.pdf)]. Because *PR-A* is a known downstream target of estrogen signaling, we also assessed the expression of coregulator proteins known to play a role in increased estrogen signaling and breast cancer. We found all three members of the p160 family of SRC proteins, SRC-1 (*p* = 0.001), SRC-2 (*p* = 0.003), and SRC-3 (*p* < 0.001), to be significantly up-regulated in response to lactational exposure to BPA [Figure 4; see also Supplemental Material, Figure 1 (http://www.ehponline.org/members/2009/11751/suppl.pdf)]. Finally, a correlation has been documented between SRC expression and expression of the erbB family of tyrosine kinases. Although we noted no significant differences for erbB1 (epidermal growth factor receptor) or erbB2 (neu), we found a significant increase in erbB3 expression (*p*= 0.01).

## Discussion

We report for the first time that oral exposure of rats to BPA via lactating dams significantly reduced tumor latency and increased tumor multiplicity in offspring in a dose-dependent manner in a DMBA model of rodent mammary carcinogenesis. In the design of these studies to determine if BPA would predispose for mammary cancer, our first considerations were model, route and timing of exposure, and dose.

Although no model is perfect, we chose rats because of the reasonable similarity in mammary gland development and use of the chemically induced mammary cancer model for tumorigenesis studies (Russo IH and Russo 1978; Russo et al. 1990). Because humans are exposed to BPA primarily by ingestion, we treated rats orally as opposed to injections or subcutaneous implants. This takes into consideration metabolism and disposition. Because timing of exposure is important to target organ toxicity and mechanism of action, we selected the neonatal/prepubertal period for these studies. In choosing the doses, we took into consideration *a*) the reports of estimated exposures of preschool children BPA at 52–74 ng/kg bw/day (Wilson et al. 2007); *b*) that the European Commission estimated BPA exposure in infants (3–6 months of age) at 0.2–8.3 μg/kg bw/day (European Food Safety Authority 2006); and *c*) that the European Union estimated exposure in infants (1–6 months of age) at 7–8 μg/kg bw/day (European Commission 2003). Accordingly, we chose to treat the dams with a low dose of BPA (25 μg/kg bw/day, 50% of the U.S. EPA’s daily oral reference dose of 50 μg/kg bw/day) and a dose that was 10-fold higher than the low dose (250 μg/kg/day). Furthermore, we exposed the offspring to significantly less BPA than the dose administered to the dams because of dilution factors resulting from maternal and fetal metabolism and disposition, lactation, and number of offspring. Although the actual blood and tissue concentrations have yet to be determined in our animals, we surmise that the effects reported in the present study are a result of low exposures to BPA. Likewise, it is possible that the effects of BPA could be on the dam, which could alter hormones and composition of the milk and consequently the offspring, issues that we have not investigated.

Our mammary cancer findings are supported by other *in vivo* studies. Durando et al. (2007) showed that prenatal exposure to BPA (via an osmotic pump, which equates to subcutaneous administration) coupled to a subcarcinogenic dose of *N*-nitroso-*N*-methylurea resulted in an increased percentage of preneoplastic and neoplastic lesions in the mammary gland. Similarly, Murray et al. (2007) found that exposure to BPA during the prenatal period through an osmotic pump resulted in the development of carcinoma *in situ* in the mammary gland even in the absence of an additional carcinogen. Although these studies used different periods of exposure, route of administration, and end point, they still provide support for our findings and add to the growing literature that implicates a detrimental role for early BPA exposure in mammary cancer susceptibility.

Our cell proliferation and apoptosis studies showed that no significant effects stemmed from direct BPA exposure (at 21 days of age). However, delayed effects did occur. At 50 days of age, > 4 weeks after discontinuing treatment with BPA, cell proliferation was significantly increased, and apoptosis was significantly decreased, in the mammary glands of rats lactationally exposed to BPA. Females lactationally exposed to BPA had a proliferation to apoptosis ratio twice that of control rats in the structures most susceptible to carcinogenesis in the mammary gland, the TEBs (Russo J and Russo 1978). Subsequent Western blotting analysis revealed that a key regulator of apoptosis, Akt and the biologically active pAkt, was significantly up-regulated. These findings were not unexpected given that both prenatal and perinatal exposures to BPA in rodents (via osmotic pumps) have been reported to cause decreased apoptosis in the mammary gland (Durando et al. 2007; Munoz-de-Toro et al. 2005). With the balance of cell turnover disrupted so severely, such a situation could provide an environment more susceptible to exposure to a secondary carcinogen, such as DMBA. The significant increase in proliferation observed in our study, but not in other studies, may be a function of differing BPA exposure schemes or routes of administration (i.e., postnatal vs. prenatal exposures, oral administration vs. subcutaneous implants, dose, etc.) that can affect metabolism and disposition.

By most accounts, BPA’s mechanism of action is thought to revolve around its weak estrogenic activity. Hence, we evaluated the effects of lactational exposure to BPA on sex steroid receptors in the mammary gland using Western blot analysis. Although we observed no significant change for ER-α or PR-B, we observed a significant increase for PR-A. This finding is supported by the work of Munoz-de-Toro et al. (2005), who found an increased percentage of cells expressing PR in the mammary gland after perinatal BPA treatment. PR has been highly implicated in breast cancer by functioning as the nuclear receptor for the sex hormone progesterone. Recent work has indicated that the two isoforms of PR function in distinct manners and are induced by differing stimuli. PR-A is the predominant isoform expressed in the pubertal, virgin mammary gland and is vital to side branching (Aupperlee et al. 2005). Studies of transgenic mice engineered to overexpress PR-A have demonstrated abnormalities in the mammary gland, including the development of hyperplastic lesions with a disorganized basement membrane and reduced cell-to-cell adhesion (Shyamala et al. 1998). Interestingly, PR-A has also been reported to be induced by estradiol in Sprague Dawley rats (Kariagina et al. 2007).

To ensure that increased expression of PR-A was not a result of lactational BPA causing increased concentrations of circulating estradiol, we measured hormone concentrations in rats at 50 days of age. We found no significant difference between the BPA-exposed and control groups, confirming that sex hormone concentrations were not altered by lactational BPA exposure. However, no significant changes that we observed manifested before the onset of puberty. One potential explanation is that early exposure to BPA increased the sensitivity of the mammary gland to the hormones of puberty. We found increased cell proliferation and PR-A expression coupled with decreased apoptosis long after the original exposure to BPA ceased. It is possible that early BPA exposure imprints or sensitizes the rat to the effects of postpubertal hormone. Then, as the concentrations of circulating sex hormones increase postpubertally, the indirect effects of BPA manifest. This theory has recently been suggested by Wadia et al. (2007), who showed that ovariectomized CD-1 mice prenatally exposed to BPA displayed a significantly heightened response in the mammary gland after an estrogenic stimulus.

A potential mechanism involved in a heightened response to estrogen is through SRC proteins. SRCs function to accelerate hormone-bound nuclear-receptor–induced transcription through weak histone acetyltransferase activity and by recruiting chromatin modification enzymes to the promoter/enhancer regions of target genes. The endogenous milieu of coregulator proteins in the target tissue are thought to have great impact on the activation of nuclear receptors, such as ER and PR, and be the basis of chemotherapeutic resistance and tissue-specific function of selective ER modulators (Schiff et al. 2003). We found the expression of the p160 family of SRC proteins (SRC-1, SRC-2, and SRC-3) to be dramatically increased at 50 days of age after lactational exposure to BPA.

Descriptions of SRC activity, as reported through transgenic mouse models, provide a close correlation with many of the reported effects of early exposure to BPA. Alterations in morphology, such as decreased side branching, decreased ductal elongation, and increased alveologenesis, have been reported in mice deficient in SRC-1 and SRC-3 (Xu et al. 1998, 2000). It seems plausible that increased expression of the SRCs in the mammary glands, as reported in our study, would confer a greater incidence of side branching and alveologenesis. Indeed, mice overexpressing SRC-3/AIB1 showed a greater degree of ductal branching (Torres-Arzayus et al. 2004). Three independent reports on early BPA exposure have described similar morphologic abnormalities, such as increased side branching and alveologenesis, in the mammary gland (Markey et al. 2001, 2005; Munoz-de-Toro et al. 2005). Knocking out SRC-3/AIB1 in mice has been shown to confer resistance to DMBA-induced mammary cancer, resulting in an increased tumor latency, reduced multiplicity, and slower growth rate (Kuang et al. 2005). Our results demonstrate that lactational BPA exposure results in a significant increase in DMBA-induced mammary cancer susceptibility at a time when the local environment of the mammary gland has drastically up-regulated expression of all of the SRCs. Likewise, transgenic mice engineered to overexpress SRC-3 exhibit an increased susceptibility to mammary cancer, developing mammary hypertrophy, hyperplasia, and malignant tumor formation after a long latency period (Torres-Arzayus et al. 2004). Prenatal BPA has been shown to cause an increase in premalignant and/or malignant lesions in the mammary glands of rats during adulthood when given alone or in concert with a sub-carcinogenic dose of *N*-nitroso-*N*-methylurea (Durando et al. 2007; Murray et al. 2007). SRC-3/AIB1-deficient mice also show reduced Akt activity, whereas SRC-3/AIB1–over-expressing mice show increased Akt activation and reduction in apoptosis in the mammary gland and in mammary tumors (Kuang et al. 2005; Torres-Arzayus et al. 2004). We report here that lactational BPA is able to up-regulate Akt expression/activation and down-regulate apoptosis in the mammary gland during adulthood. Finally, Xu et al. (2007) described perinatal exposure to BPA in Sprague Dawley rats causing a significant increase in *SRC-1* mRNA and protein in the hippocampus of male pups.

A relationship has been established in the literature between the SRCs, HER2/erbB2, and the Akt signaling pathway. One study reported SRC-1 to be overexpressed in 46% of HER2-positive breast tumors but in only 6% of HER2-negative breast tumors (Fleming et al. 2004). Bouras et al. (2001) reported a positive correlation between the expression of SRC-3, HER2, and p53. Although HER2 breast tumors are frequently cited as resulting in shorter disease-free survival, coupling HER2 overexpression with SRC-3 overexpression has been reported to worsen the prognosis further, adding a greater risk of refractory disease (Kirkegaard et al. 2007; Mc Ilroy et al. 2006; Osborne et al. 2003). SRC-3/AIB1 has also been shown to control the activity of both erbB1 and HER2 (erbB2) (Lahusen et al. 2007). Down-regulating erbB3 through small interfering RNA reduces Akt activity (Liu et al. 2007). These results suggest that coactivator proteins may serve as essential mediator proteins, linking crosstalk between growth factors and sex steroid signaling pathways.

Although we noted no changes between treatment groups in the expression of erbB1, erbB2, or erbB4, we found a significant up-regulation for erbB3. erbB3, despite lacking a catalytic domain, has been reported to be the preferred dimerization partner for erbB2. This pairing has also been reported to induce the most mitogenic response that preferentially signals through the Akt pathway (Karamouzis et al. 2007). Although overexpression of erbB3 alone may induce mammary carcinogenesis, overexpression of both erbB2 and erbB3 has been observed frequently in a transgenic mouse model that overexpresses the wild-type erbB2 protein, and in human breast tumor samples (Karamouzis et al. 2007). This dual over-expression has been linked to more aggressive disease and increased development of drug-resistant cancer. Thus, although we did not observe the up-regulation of erbB2 at this specific time point (50 days of age), an up-regulation of erbB3 could provide ample access to a binding partner for erbB2 and provide a local environment more prone for cancer development.

## Conclusion

The data we present here provide the first evidence that maternal exposure to BPA during lactation decreased time to first tumor latency and increased the number of DMBA-induced mammary tumors in female offspring. We conclude that increased cell proliferation and decreased apoptosis at the time of DMBA administration play a vital role in BPA’s mechanism of action. Furthermore, we believe that these effects are, at least in part, mediated through the increased expression of erbB3 and the p160 family of SRCs (SRC-1, SRC-2, and SRC-3/AIB1) and increased Akt activity. Interestingly, the published observations of early exposure to BPA overlap well with the reported phenotypes of transgenic mouse models of SRC overexpression and counter models of SRC deficiency. However, not only do the alterations reported here provide an explanation of enhanced mammary carcinogenesis after lactational treatment with BPA, but they also warn of the potential for developing more aggressive and refractory forms of breast cancer. In coculturing nonmalignant, random periareolar fine-needle aspirates from the contra-lateral breast of women with breast cancer, Dairkee et al. (2008) found that physiologic doses of estrogen and progesterone coupled to BPA produced a gene signature profile that was associated with breast tumors with high pathologic grade, greater tumor size, and shorter disease-free patient survival. Increased expression of erbB2, erbB3, the SRCs, and Akt activation and have been linked with tamoxifen resistance (Bouras et al. 2001; Kirkegaard et al. 2007; Mc Ilroy et al. 2006; Osborne et al. 2003). In breast cancer patients, high SRC-3/AIB1 expression is associated with HER2 overexpression, tamoxifen resistance, and higher mortality. If these effects found in rodents carry over to humans, even small, seemingly harmless exposures of BPA early in life could be detrimental and play a role in increased breast cancer susceptibility as well as the ability of a tumor to become resistant to therapy.
